# Cooperation between gatekeepers in sickness insurance – the perspective of social insurance officers. A qualitative study

**DOI:** 10.1186/1472-6963-8-231

**Published:** 2008-11-07

**Authors:** Carina A Thorstensson, Jenny Mathiasson, Barbro Arvidsson, Anders Heide, Ingemar F Petersson

**Affiliations:** 1 The MORSE project Musculoskeletal Research Centre, Department of Orthopedics, University Hospital, Lund, Sweden; 2 Research and Development Centre, Spenshult, Halmstad, Sweden; 3 School of Social and Health Sciences, Halmstad University, Halmstad, Sweden; 4 Faculty of Nursing Science, Gjövik University College, Gjövik, Norway; 5 Swedish Social Insurance Agency, Malmö, Sweden

## Abstract

**Background:**

Objective was to describe variations in how social insurance officers conceive the cooperation with the health care in their daily work with sick leave.

**Methods:**

Fifteen social insurance officers (SIOs) working with administration of sickness benefits were interviewed. They were purposefully recruited to represent different parts of the social insurance office organization, different ages, gender, education, and work experience. The interviews were audio-recorded, transcribed verbatim and analyzed using phenomenographic approach.

**Results:**

11 women and 4 men, aged 25–65, with a work experience ranging from 1–40 years were interviewed. Three descriptive categories embracing eleven subcategories emerged: 1) *Communication channels *included three subcategories; *to obtain medical opinions, to hold meetings with actors involved, to experience support functions*; 2) *Organizational conditions *included five subcategories; *to experience lack of time, to experience problems of availability, to experience lack of continuity, to experience unclear responsibility, to experience ongoing change*; 3) *Attitudes *included three subcategories; *to conceive the attitudes of the physicians, to conceive the attitudes of the patients, to conceive the attitudes of the SIOs*.

**Conclusion:**

Personal communication was described as crucial to ensure a more efficient working process. The personal contact was obstructed mainly by issues related to work load, lack of continuity, and reorganisations. By enhancing and enabling personal contact between SIOs and health care professionals, the waiting times for the sick-listed might be shortened, resulting in shorter periods of sick-leave. Issues around collaboration and communication between gatekeepers need to be recognized in the ongoing work with new guidelines and education in insurance medicine.

## Background

Cooperation problems between different actors within the sickness absence process have been reported to cause long periods of sick leave, often including passive times of waiting and unequal treatment of patients [1-3]. The Swedish welfare policy and legislation allows for partial economic compensation for loss of income due to sick leave and reduced work capacity caused by medical conditions. Towards the end of the 20th and the beginning of the 21st century a sharp rise in the number of employees on long-term sick leave was seen in Sweden as well as in other western European countries, together with a tendency towards longer periods of sickness absence, with consequences for the society as well as the individual [4-7].

The social insurance system in Sweden is administrated by the National Social Insurance Board, and largely executed by social insurance officers (SIOs). They make their decisions regarding entitlement to sickness benefits based on medical assessments and sickness certificates issued by physicians [8]. The certificate should contain information on the medical diagnosis (ICD code), duration and degree of work capacity reduction, working situation of the patient, prognosis for recovery and rehabilitation recommended [8]. According to the Swedish National Insurance Act the social insurance office has to cooperate with the health services, the employers and other important actors in the rehabilitation process on a local, regional and central level, to support the individual needs of the patient [9,10]. The SIOs and the physicians are thus important "gatekeepers" in the sickness absence process. Their performance and cooperation is crucial both in terms of people's security and welfare [11].

The extent and the high costs of sick leave in Sweden have aroused a new research area. Interaction between involved actors in the sickness absence process has been focus in several recent publications [1,11-15]. Most studies on sick listing focus on physicians, and the perspective of SIOs is less often explored. The aim of the present study was to describe variations in how SIOs in southern Sweden conceive the cooperation with the health care in their daily work with sick leave.

## Methods

### Informants

The social insurance office in the region of south Sweden, where the study was conducted, was at the time of interviews organized in five different geographic areas. Within each geographic area the work on administration of sickness benefits and rehabilitation was divided into two separate work processes: (1) early assessment of new cases; (2) advanced investigation and rehabilitation. For this study the managers of the different units received structured oral or written information. Three informants from each of the different areas were recruited after informed consent. The informants were strategically chosen to represent all geographic areas, and both working processes, and also various ages, gender, levels of education, and number of years working as a SIO. All fifteen SIOs were interviewed during spring 2007. Ethical approval was obtained from the Regional Ethics Committee, Medical Faculty, Lund University (187/2007).

### Interviews

The participants were interviewed using an open, semi-structured interview guide. Interviews took place at a place chosen by the participant, and each interview started with an open question on how the cooperation with health care in their daily work was conceived of by the informant. Aspects brought up by the interviewee were probed in more depth, and each interview lasted 30–90 minutes. Two pilot interviews were conducted to test the relevance of the interview guide. No changes were made and the two pilot interviews were included in the analysis. The focus of this study was cooperation between SIOs and health care only. Other important collaborators in sickness absence process, like employers and job agencies, were not targeted. All interviews were conducted by a researcher with experiences from work as a SIO (JM). The interviews were audio-taped and transcribed verbatim.

### Analysis

The analysis was conducted using a phenomenographic approach. Phenomenography focuses on describing variations in how people conceive and experience a phenomenon, and it distinguishes between the actual state of something and how it is conceived. The method was developed in educational research and first described by Marton [16].

The analysis began with reading and re-reading of the transcripts and listening to the recordings to obtain an overview of the data. During the data reduction process conceptions corresponding to the aim of the study were identified and included in further analysis. The conceptions were then grouped together into content-related sub-categories. At this stage the number of categories was large. Similarities and differences between sub-categories were identified, and categories representing a variation within the same theme were grouped together and further assigned to a more general descriptive category. The relationship between the part and the whole was scrutinized, and this process continued until the descriptive categories were different in context and meaning and corresponded to the context. The sub-categories and descriptive categories were labelled in order to highlight their essence, and quotes were chosen to illuminate the range of conceptions within each category. Analysis and results were cross-checked by authors with experience from qualitative research (BA, CT), and discussed between all authors during the process.

## Results

The informants were 11 women and 4 men, aged 25–65. Four worked with early assessments, 7 with further consideration of long-term sick-listed with employment, and 4 were involved in further consideration of unemployed on long-term sick-leave. Their experience from working as a SIO ranged from one year to more than 40 years. Four of them had pre-university education, 4 had less than two years of university education, and 7 had two or more years of university education.

Three qualitatively different descriptive categories and eleven subcategories emerged from the analysis. The descriptive categories were *communication channels*, *organizational conditions *and *attitudes*, described in more detail below.

### Communication channels

Different channels of communication between the health care and the SIOs were described by the informants, and three different sub-categories of communication channels were identified; *to obtain medical opinions*, *to hold meetings with actors involved*, and *to experience support functions*.

#### To obtain medical opinions

Certificates lacking vital information, like proper medical diagnoses and assessments of the patients work capacity, were described as problematic by the informants. Deficient certificates generated a great deal of extra work and were conceived of as source of conflict with the physicians. To wait for supplementary assessments delayed the decision process and was described as inefficient and time-consuming.

*There is a risk that the sick-listed persons do not get their sickness benefit due to a lousy certificate. We try to improve the process, we try to inform about it [the importance of a complete certificate] but the importance of the certificate is not clear to the health care*. (Ip 12)

On the other hand when a certificate was well issued this was described as leading to a better collaboration considering all aspects of the cooperation.

*It becomes a better contact if I see a sickness certificate where it says clear and articulate that this is how it is. Then I understand the situation and I understand what to do, and our cooperation gets much easier from the start*. (Ip 04)

#### To hold meetings with actors involved

Joint meetings with the sick-listed, the employer and the doctor were described as one of the most important channels of communication, as well as a significant factor for shortening a period of sick leave. Meetings enabled a joint planning and enhanced the mutual understanding for each others work. At the same time great difficulties in arranging meetings was reported and several informants said that joint meetings where seldom or never held with physicians present.

*If we talk about joint meetings, it's really hard to get a visit from a doctor. We try to arrange that, and hold the meetings there [where the doctors are], but it's very rare...//it's most often just the sick-listed and the employer because it's so hard for the doctor to find the time*. (Ip 04)

#### To experience support functions

The informants described several types of functions aiming at supporting the cooperation in different ways. The formation of the support differed from area to area. In some areas one or two SIOs where allocated to work full time on the dialogue with the health service. In other areas insurance doctors were appointed to educate other physicians in the area, and special information campaigns were held. Several informants said that they had noticed a difference in the certificates and attitudes amongst the physicians after such educations or campaigns.

*There was a big effort some two or three years ago when all primary care centres in this region were contacted and educated during one day. Education in social insurance so to speak, to all doctors. I think the understanding of the role of the social insurance office has improved among doctors. Not in every case, but it's getting better and better*. (Ip 10)

In some areas individual SIOs working in the regular parts of the organization were appointed as contact persons towards a certain clinic or unit within the health care. The conceptions of the system with contact persons differed between the informants – some found them useful while others described them as an unnecessary extra link.

*It is an unrewarding task, because you are the contact person for the whole social insurance office and you are not discussing your own agenda*. (Ip 05)

### Organizational conditions

Various organizational conditions affecting the cooperation were described. Five sub-categories emerged; *to experience lack of time*, *to experience problems of availability*, *to experience lack of continuity*, *to experience unclear responsibility*, *to experience an ongoing change*.

#### To experience lack of time

This sub-category held descriptions of problems generated from the physicians having very little time to spend on the process of each patient, as well as their own experience of lack of time. Physicians were conceived of as having insufficient time to contact the informants, to attend joint meetings, and to make a proper assessment of the patient's capacity to work.

*They [the physicians] are supposed to see a number of patients every day, and they have 15 minutes per patient including administration/.../there is not much room for anything else that is at all time consuming*. (Ip 06)

The informants also described how they themselves, because of time pressure, did not contact physicians and other parties as much as they would have needed.

*With a work load more adapted to the time available, I do believe that every SIO would have a very, very much improved collaboration with everyone involved. That is the issue. It is not possible to handle the number of cases the way they are supposed to be handled. I am convinced that this is the first thing that needs to be corrected. The contacts would improve, and the quality and results of our work would be a lot better*. (Ip 07)

#### To experience problems with availability

Within this sub-category the informants described difficulties in getting in contact with the physicians. The informants described how most social insurance offices had a special phone number for physicians to facilitate the contact on their behalf. The SIOs, on the other hand, had no direct telephone numbers where the physicians could be reached. Instead they had to call secretaries or receptionists to leave a message, often after waiting in long telephone queues, and it was common that their calls were never returned by the physician. As a result the SIOs avoided contacting the physicians by phone, or arranging meetings. This lead to more usage of paper forms, despite the fact that a personal contact was considered as an important part of good cooperation.

*Occasionally I call, of course, and I have to leave a message at the secretary. This leaves us in a vicious circle, they call back when I'm not in, and I call back when they can't talk. Ironically, doctors and SIOs cannot call each other. We never seem to reach each other*. (Ip 15)

#### To experience lack of continuity

This sub-category described a frequent staff turnover within the two organizations, and this was conceived of as an obstacle to cooperation.

*I've never experienced such a large turnover of SIOs as it is right now. It's like playing soccer in a team where the midfield players are exchanged every third...it's weird./.../The only and immediate consequence of this is longer periods of sick leave, that's the only consequence. Of course it effects the cooperation with health care*. (Ip 02)

*And the patients see a lot of different physicians. If you send a request it's unlikely that the physician will do anything because the patient will see another physician the next time*. (Ip 14)

Well developed and continuous contacts between the SIOs and the physicians were considered to be important factors for a fruitful cooperation and better mutual understanding between the two authorities.

#### To experience unclear responsibility

The responsibility for the assessment of the work capacity of the sick-listed was described as unclear. The SIOs use the physicians' sickness certificates as a basis for their decisions regarding entitlement to insurance benefits. This was sometimes conceived of as leading to physicians dumping their problems over on the social insurance office. Rather than taking a discussion with the sick-listed patient the physicians would issue a vague certificate, knowing that the sick-leave might not be approved by the insurance office, and in this way letting the SIOs take the "blame".

*The consequence is indistinctness, especially towards the sick-listed patient. This means that their view upon the SIOs working on the office might be distorted. And it can have a large impact on the personal economy of the sick-listed, at the same time as my competence is questioned, and it may even cause debate in newspapers*. (Ip 03)

#### To experience ongoing change

The informants described how their work had become more structured, how they now followed the regulations more strictly and questioned the physicians' assessments more often than before. They conceived that they made stricter assessments, more rigorous investigations and more suspensions.

*We have a lot more suspensions of sickness benefits during ongoing sick-leave today than we had just a year ago. This is all due to the new guidelines we are supposed to follow at work. The rules and laws are not changed, but previously we were allowed to interpret them slightly different, and be more generous in our assessment, and this has been regulated*. (Ip 07)

Concurrently the informants experienced that they had less dialogue and a less active cooperation with the physicians than before and that joint plans were less often constructed. One informant reckoned this as a result of an increased focus from the insurance office management on the cooperation with employers and job centres during the last years.

### Attitudes

The cooperation with health care was conceived of as being affected by attitudes amongst the *physicians*, the *patients *and *the SIOs*. The attitudes towards sick leave differed between the health care service and the social insurance office, with the attitude of health care described as more caring, while the SIOs had a stronger focus on regulations.

#### To conceive the attitudes of the physicians

The physicians' attitudes towards sick leave, and their knowledge about the social insurance system, were conceived of as important factors for cooperation and also as determinants for a successful rehabilitation. The attitudes were described as varying greatly between the individual physicians. Some physicians were conceived of as making strict medical assessments, while others were described as taking on a social responsibility and issue certificates because of family or work related problems. In some cases physicians were described as working hard getting patients back to work, while others were conceived of as more passive and routinely prolonging the periods of sick leave.

*Some physicians really make an effort to get into the diagnosis and they have a plan. They don't just prolong but question the sick leave, state what treatments have been done in the certificate and use a stepwise progress to get people back to work*. (Ip 4)

*There are still some doctors who are negative towards the social insurance office and negative towards our way of working. Their attitude is that if you're ill you're ill, no matter what*. (Ip 01)

Several informants expressed a recent change in attitude of many physicians, from a more passive to a more active approach. Physicians were now more often conceived of as having a better understanding for the negative effects from a passive absence from work.

#### To conceive the attitudes of the patients

The individual motivation of the patient was described as crucial to the results from the rehabilitation process in general. Some patients were conceived of as taking the sickness certificate as a guarantee for sickness benefit, causing problems in communication between the different actors within the process. One informant mentioned that a good cooperation between the social insurance office and the health service could stop less motivated patients from playing out one against the other.

*There are some [sick-listed] who don't want to, or do not gain anything from getting well. To them a good cooperation [between health care and social insurance office] is negative*. (Ip 14)

#### To conceive the attitudes of the SIOs

The importance of a positive attitude of the SIOs, and a willingness to try and create a dialogue was emphasized, and described as important to facilitate a good cooperation.

*To have a dialogue we can't just sit here in our office and use a lot of forms. I think the dialogue is crucial, that is what the work on improvements is about*. (Ip 11)

The conceptions of the professional role varied. Some informants described the SIO as a professional guard of the social insurance system, and stressed the importance of following regulations.

*We base the decision on what is written, and that has caused problems. They [the physicians] don't understand that we don't take a lousy certificate. We don't use our imagination. If it's not on the certificate, it doesn't exist*. (Ip 12)

Others conceived the SIO as a mediator, and emphasized the personal responsibility to ensure the patient did not suffer because of deficient medical certificates, short-comings in the system or problems with communication between the two authorities.

*The problem is that the sick-listed does not get the sickness benefit, and I try to find a way to avoid problems for third party*. (Ip 3)

## Discussion

This study showed that a direct and continuous contact with the physicians was considered by the SIOs to be one of the best ways of enhancing a mutual understanding and improve the cooperation between the social insurance office and health care. However, a more in depth approach revealed that direct communication rarely occurred in daily practice. Issues like irregular availability, lack of time, and ongoing changes within the organization were described as obstacles to direct contact via meetings and telephone.

SIOs had the final decision on entitlement to sickness benefits, however their decisions were based on the sickness certificates issued by physicians. Problems with deficient certificates were frequently mentioned by the SIOs in this study, and it has also been identified as a problem in relation to disability pensions [17]. The quality of sickness certificates has been found to be poor, and crucial information on medical condition and/or functional capacity was lacking in more than 70% of 2400 issued certificates in a recent Swedish study [8]. This has been recognized in other countries as well. An audit of sickness absence referrals in Scotland some years ago revealed that only 12% contained information about job tasks [18]. The consequences are that certificates need to be returned for completion, resulting in an increased workload for SIOs as well as prolonged processes and longer periods of sick-leave [8,19]. To improve the quality and content of sickness certificates, supportive functions and educational interventions had been introduced and were perceived as successful by some SIO's. However, the impact on sick listing and work related outcome was not mentioned. Earlier studies have shown that interventions aiming at facilitating joint comprehensive actions among gate-keepers in the sick-listing process, and changes in regulations and administration routines, improved the certificates but had low impact on length of sick leave and the number of new cases on sick leave [15,20].

According to sickness certificates used in Sweden until 2007, work capacity was assessed in relation to "objective findings" of the disease (National Social Insurance Board 1994). In reality, factors other than the medical disease are highly related to work capacity and sick-listing, such as age, gender and psychosocial situation of the patients, but also attitudes and interactions between different actors in the process [2,21]. The different attitudes towards sick leave among different actors has been recognized as a source of communication problems [17], and was mentioned in this study as present both between and within organisations. The sick-listing process is also influenced by other actors such as employers, employment agencies or jobcentres, and the sick-listed. A recent study showed that problems due to unemployment and sick-leave often are shifted on to a medical problem [12]. A joint cooperation between all the different actors in the sickness absence process, included the sick-listed person, is important to optimize measures for the individual and to reduce the length of time on sick-leave to a minimum. However, this was not within the scope of this study.

The daily work of SIOs has been described earlier, and it is striking that not much seem to have changed seen from the perspective of SIOs during the past 10 years [19]. However, a change towards a more direct approach, from accepting to questioning physicians recommendations, can be distinguished over time [19,22]. Informants in this study described their role today as more structured compared to some years ago. The change towards a more structured and controlled sickness absence legislation has also been seen over the past decades in Denmark. The Danish system is based on regular assessments of work capacity by the municipal officer entitled to the sickness absence process. Focus is on the return-to-work-process, and the sick-listed is still an important actor, but the autonomy of the individual has been reduced [21].

The continuously ongoing changes within the social insurance office and the health care were sometimes conceived of as a direct obstacle to personal contact. The lack of continuity, lack of availability, lack of time, and lack of personal contact forced more use of paper forms, and prolonged the passive waiting times for the sick-listed. The lack of personal contact has also been described as frustrating from the physicians perspective [22], and closer cooperation between different actors has been suggested, not only by the SIOs in this study, as a possible strategy to improve the work rehabilitation process [23]. A process to further develop the communication between the health care and the SIOs in the area of musculoskeletal disorders has been initiated in the southern parts of Sweden .

The possibility to formulate and test a new hypothesis within phenomenography is restricted compared to other qualitative methods, which limits the interpretation of the results into a wider context. However, the phenomenographic approach was considered relevant to describe the experiences and conceptions of the SIOs. It can be argued that the researcher can influence the interview and analysis process. On the other hand, a thorough knowledge of the field is crucial to be able to probe in more depth themes that are brought up, and also to understand the concepts described by the informant. The risk of influence is also minimized by triangulation, i.e. the results are discussed and agreed among several researchers with different professional background, and with qualitative as well as quantitative experience. This study is based on 15 interviews only. The small number of informants included can limit the ability to generalize the results to a wider population. However, the informants were purposively chosen to represent different parts of the social insurance office organization, as well as age, gender, education, and work experience, to ensure as a varying description as possible.

## Conclusion

Personal communication was described as crucial to ensure a more efficient working process. The personal contact was obstructed mainly by issues related to work load, lack of continuity, and reorganisations. By enhancing and enabling personal contact between SIOs and health care professionals, the waiting times for the sick-listed might be shortened, resulting in shorter periods of sick-leave. Ongoing work with new guidelines and education in insurance medicine need to recognize and aim at improving the issues around collaboration and communication between gatekeepers.

## Competing interests

The authors declare that they have no competing interests.

## Authors' contributions

CT and JM contributed equally to the work. CT participated in analysis and interpretation of the data, and drafted the manuscript. JM participated in the design of the study, carried out the interviews, analysis and helped in drafting the manuscript. BA participated in the design, analysis and interpretation of data, and critically revised the manuscript. AH participated in design and critically revised the manuscript. IP participated in the design of the study, and critically revised the manuscript. All authors read and approved the final manuscript.

## Pre-publication history

The pre-publication history for this paper can be accessed here:


